# Do psychophysiological responses to competitive anxiety predict competition outcomes? A pilot study in elite female karate athletes

**DOI:** 10.1016/j.jsampl.2025.100132

**Published:** 2025-12-22

**Authors:** Maryam Kenshloo, Behrouz Golmohammadi, Vahid Khashani, Amir Hossien Mehrsafar

**Affiliations:** a Division of Motor Behavior, Department of Physical Education and Sports Sciences, Faculty of Humanities, Semnan University, Semnan, Iran; b Division of Sport Psychology, Department of Physical Education and Sports Sciences, Faculty of Humanities, Semnan University, Semnan, Iran

**Keywords:** Pro-inflammatory markers, Competitive anxiety, Salivary cortisol, Female athletes, Karate

## Abstract

Evaluating psychophysiological markers is key to understanding the allostatic burden of official competitions. This study investigated the associations between responses to competitive anxiety and salivary biomarkers—cortisol (sCort), interleukin-6 (sIL-6), and tumor necrosis factor-alpha (sTNF-α)—in elite female karate athletes during competition. Thirty participants (mean age = 21.8 ± 3.4 years) completed the Persian version of the Competitive State Anxiety Inventory-2 Revised (CSAI-2R) approximately 1 h prior to competition to assess cognitive anxiety, somatic anxiety, and self-confidence. Saliva samples were collected 30 min before the competition to quantify biomarker levels. Significant positive associations were observed between sCort and both somatic and cognitive anxiety (p < 0.05), whereas no significant associations were detected between anxiety subscales or self-confidence and sIL-6 or sTNF-α (all p > 0.05). Multiple regression results showed that cognitive and somatic anxiety significantly predicted sCort (p < 0.05), while none of the CSAI-2R variables significantly predicted sIL-6 (p > 0.05) or sTNF-α (p > 0.05). Logistic regression analysis revealed that somatic anxiety significantly predicted competition outcome (p < 0.05), whereas other variables did not. These findings highlight the potential utility of monitoring somatic anxiety as a marker of competitive anxiety in elite female karate athletes.

## 1 Introduction

Competitive anxiety is a multidimensional negative emotional state elicited in response to athletic situations perceived as threatening or stressful, and is characterized by three interrelated dimensions: cognitive appraisals (e.g., worry, negative performance-related thoughts, and impaired concentration), physiological arousal (e.g., elevated heart rate, muscle tension, and somatic restlessness), and behavioral manifestations (e.g., irritability, agitation, and alterations in interpersonal conduct) [1]. Research shows that karate athletes commonly experience elevated anxiety before competitions, which can impair performance [2]. In stressful situations, subcortical brain regions such as the hypothalamus and amygdala activate the hypothalamic–pituitary–adrenal (HPA) axis, leading to increased cortisol secretion. Elevated salivary cortisol (sCort) before and during competition has been linked to performance impairments across various sports [3], and a strong association between competitive anxiety and sCort levels has been consistently reported [4,5]. Competition-induced stress activates the immune system, resulting in complex interactions among the nervous, endocrine, and immune systems [6]. Acute psychological stress enhances immune responses, with pro-inflammatory cytokines such as IL-6 and TNF-α playing central roles in the regulation of emotion, behavior, and cognitive processes [7]. Elevated salivary levels of IL-6 (sIL-6) and TNF-α (sTNF-α) are commonly observed in response to psychosocial stress [8], and clinical studies have shown that anxiety is associated with higher levels of these cytokines [9]. While the role of sCort in stress and performance is well established, the contributions of sIL-6 and sTNF-α in the context of competitive anxiety remain underexplored. To date, no study has simultaneously examined the interplay among salivary cortisol, IL-6, TNF-α, and competitive anxiety in elite female karate athletes. Addressing this gap, the present study investigates the associations among competitive anxiety, salivary biomarkers (sCort, sIL-6, and sTNF-α), and competitive outcomes (win/loss) in elite female karate athletes during an official competition. Specifically, it examines whether dimensions of competitive anxiety predict salivary biomarker levels and whether these psychological and physiological indicators predict performance outcomes.

## 2 Method

### 2.1 Participants

The sample size was determined using a priori power analysis in G∗Power (v3.1.9.7), which indicated a required sample size of 28 participants based on a medium effect size (f^2^ = 0.15), *α* = 0.05, and power = 0.80. To enhance statistical reliability, 30 elite female karate athletes (mean age = 21.8 ± 3.4 years; BMI = 24.8 ± 1.9 kg/m^2^) from the Iranian national team camp were recruited, comprising national (46.7 %) and international (53.3 %) competitors. All participants were healthy, with no history of mental or physical illnesses, as confirmed by both self-report and medical records. The study was approved by the Research Ethics Committee of the Iran Sports Science Research Institute (number ID: IR.SSRC.REC.1402.128) and conducted in accordance with the Declaration of Helsinki. All participants provided written informed consent and received no compensation for their involvement.

### 2.2 Psychometric assessments

#### 2.2.1 Competitive State Anxiety Inventory-2 (Revised version)

The revised Persian version of the Competitive State Anxiety Inventory-2 (CSAI-2R) was used to assess the multidimensional components of competitive anxiety [1]. It includes 16 items across three subscales: somatic anxiety (6 items), cognitive anxiety (5 items), and self-confidence (5 items), rated on a 4-point Likert scale (1 = “Not at all” to 4 = “Very much so”). Higher scores indicate greater intensity of the respective psychological states. The Persian version of the CSAI-2R has been validated for use with Iranian athletes, with evidence supporting its reliability and factorial validity [10]. Athletes completed the paper-based inventory 1 h prior to the competition.

### 2.3 Physiological assessment

Prior to saliva collection, participants adhered to standardized pre-sampling protocols, including abstinence from food, drink, and oral hygiene. Saliva was collected via passive drooling into single-use cups over 2 min, immediately centrifuged at 3000 rpm for 10 min and stored at −20 °C until analysis. Salivary cortisol (nmol/L) was measured using an ELISA kit with a sensitivity of 0.1 nmol/L and an intra-assay coefficient of variation (CV) of less than 8 %. sTNF-α and sIL-6 (ng/L) were analyzed using sandwich ELISA kits with a detection limit below 10 ng/L and an intra-assay CV of less than 10 %.

### 2.4 Procedure

**Fig. 1:**
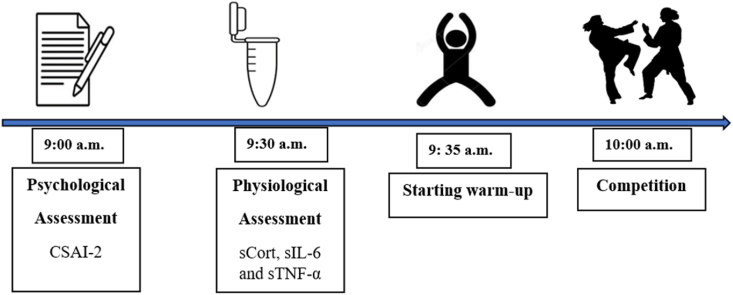
Schematic representation of the study setting. Participants completed the CSAI-2 60 min before the competition. Besides, saliva sample was collected 30 min before competition Note: CSAI-2R, Competitive State Anxiety Inventory-2 Revised version; sCort, Salivary Cortisol; sIL-6, salivary interleukin-6, sTNF-α, salivary tumor necrosis factor-alpha.

The study was conducted in June 2023 during the internal selection competition for the women's national karate team, providing a high-pressure, real-world context. Competitions adhered to World Karate Federation (WKF) regulations. One week prior to the competition, during a stress-free condition, baseline saliva samples (2.5 mL) were collected from athletes. On competition day, athletes completed the CSAI-2R approximately 1 h before the event (∼9:00 a.m.), and saliva samples for sCort, sIL-6, and sTNF-α were collected 30 min later (∼9:30 a.m.). Warm-ups began afterward, with competition starting at 10:00 a.m. Competition outcomes were recorded as binary win/loss results. A detailed timeline is presented in Fig. 1.

### 2.5 Statistical analysis

Data were screened for normality and outliers, with the Shapiro–Wilk test indicating normal distribution. Paired t-tests compared sCort, sIL-6, and sTNF-α levels between competition and stress-free conditions. Pearson's correlations assessed associations between psychological variables (somatic anxiety, cognitive anxiety, self-confidence) and physiological markers. Additionally, point–biserial correlations were calculated between binary competition outcomes (win/loss) and both psychological and physiological indices to explore potential associations. Effect sizes for associations were interpreted based on Cohen's guidelines (small: *r* = 0.1, medium: *r* = 0.3, large: *r* = 0.5). Multiple regression analysis with the enter method was conducted to determine whether CSAI-2R subscale scores predicted levels of salivary biomarkers (sCort, sIL-6, and sTNF-α). To further investigate the influence of competition outcomes, a logistic regression analysis was performed, using competition outcomes (win/loss) as the dependent variable and psychological and physiological markers as independent variables, employing both enter and backward stepwise methods). Adjusted R^2^ values, odds ratios (OR), and 95 % confidence intervals (CIs) were reported to estimate effect sizes and their precision. Statistical significance was set at *p* < 0.05. All analyses were performed using IBM SPSS Statistics (version 27).

## 3 Results

sCort levels were significantly higher on the day of competition than during the stress-free condition (baseline: M±SD = 21.45 ± 1.73, competition: M±SD = 24.25 ± 4.56, t[29] = 3.22, p = 0.01, Cohen's d = 0.59). Conversely, no significant changes were observed for sIL-6 (baseline: M±SD = 2.92 ± 0.13, competition: M±SD = 2.86 ± 0.51, t[29] = −0.62, p = 0.54, Cohen's d = −0.11), or sTNF-α (baseline: M±SD = 0.66 ± 0.05, competition: M±SD = 0.67 ± 0.11, t[29] = 0.29, p = 0.77, Cohen's d = 0.05) between conditions.

Pearson's correlation revealed significant positive associations between sCort and both somatic and cognitive anxiety, but no association with self-confidence. No significant associations were found between sIL-6 or sTNF-α and any of the psychological variables (Table 1). Point-biserial correlations showed that salivary biomarkers were not significantly associated with competition outcomes (win/loss). However, both somatic and cognitive anxiety were significantly negatively associated with competition outcomes, whereas self-confidence showed no significant association (Table 1).

**Table 1:** Descriptive statistics, bivariate correlations (Pearson's r), point–biserial correlations (rpb), and summary of multiple and logistic regression analyses for predicting salivary biomarkers and competition outcomes in elite female karate athletes.

Variable	Mean (SD) or n (%)	Somatic Anxiety	Cognitive Anxiety	Self-Confidence	Competition Outcome
		r (p) [95 % CI]	r (p) [95 % CI]	r (p) [95 % CI]	rpb (p) [95 % CI]
Competition outcome	Win = 13 (43.3 %)	–	–	–	–
	Loss = 17 (56.7 %)				
Somatic anxiety (score)	10.70 (1.89)	–	–	–	−0.47∗∗ (p = 0.01) [0.13, 0.71]
Cognitive anxiety (score)	9.10 (2.48)	0.52∗∗ (p = 0.01) [0.21, 0.74]	–	–	−0.39∗∗ (p = 0.03) [0.03, 0.66]
Self-confidence (score)	14.63 (2.81)	−0.45∗ (p = 0.01) [-0.70, −0.11]	−0.48∗∗ (p = 0.01) [-0.71, −0.15]	–	0.31 (p = 0.09) [-0.60, 0.05]
Salivary cortisol (nmol/L)	24.25 (4.65)	0.53∗∗ (p = 0.01) [0.20, 0.75]	0.52∗∗ (p = 0.01) [0.19, 0.73]	−0.09 (p = 0.60) [-0.44, 0.27]	−0.28 (p = 0.13) [-0.08, 0.58]
Salivary IL-6 (ng/L)	2.86 (0.51)	−0.10 (p = 0.58) [-0.44, −0.26]	−0.08 (p = 0.65) [0.20, 0.74]	−0.12 (p = 0.49) [-0.46, 0.24]	−0.10 (p = 0.58) [-0.44, 0.26]
Salivary TNF-α (ng/L)	0.67 (0.11)	−0.01 (p = 0.94) [-0.34, 0.37]	−0.25 (p = 0.18) [-0.12, 0.56]	0.07 (p = 0.68) [-0.29, 0.42]	−0.05 (p = 0.77) [-0.40, 0.31]

Multiple regression	Salivary Biomarkers											
	Salivary cortisol				Salivary IL-6				Salivary TNF-α			
Variable	B	β	t	p	B	β	t	p	B	β	t	p
Somatic anxiety	1.07	0.43	2.41	0.02∗	−0.06	−0.25	−1.09	0.28	−0.01	−0.09	−0.44	0.66
Cognitive anxiety	0.83	0.44	2.43	0.02∗	0.02	0.13	0.55	0.58	0.01	0.41	1.81	0.08
Self-confidence	0.52	0.31	1.80	0.08	−0.03	−0.18	−0.80	0.42	0.01	0.23	1.06	0.29

Logistic regression (enter method)	Competition outcome (Win/Loss)				
Variable	B	Wald statistic	p	Odds ratio (OR)	95 % CI
Somatic anxiety (score)	−0.47	1.70	0.19	1.60	[0.79, 3.24]
Cognitive anxiety (score)	−0.21	0.74	0.38	1.24	[0.76, 2.02]
Self-confidence (score)	0.04	0.05	0.81	0.95	[0.65, 1.40]
Salivary cortisol (nmol/L)	−0.01	0.01	0.90	0.98	[0.76, 1.27]
Salivary IL-6 (ng/L)	−0.47	0.23	0.62	0.62	[0.09, 4.10]
Salivary TNF-α (ng/L)	−1.99	0.24	0.61	0.13	[0.01, 352.35]

**Logistic regression (backward stepwise)** **Step: 6**	**Competition outcome (Win/Loss)**				

Somatic anxiety (score)	−0.61	5.59	0.01∗∗	1.85	[1.12, 3.10]

Note: B: unstandardized beta; β: standardized regression weight; ∗p < 0.05, ∗∗p < 0.01, n = 30.

As shown in Table 1, the multiple regression model predicting sCort from the three CSAI-2R subscales was statistically significant (R^2^ = 0.43, adjusted R^2^ = 0.36, F[3, 26] = 6.61, p = 0.01), with somatic and cognitive anxiety emerging as significant predictors, each demonstrating moderate-to-large effect sizes. Self-confidence was not a significant predictor. In contrast, regression models for sIL-6 (R^2^ = 0.06, adjusted R^2^ = −0.04, F[3, 26] = 0.55, p = 0.64) and sTNF-α (R^2^ = 0.12, adjusted R^2^ = 0.01, F[3, 26] = 1.18, p = 0.33) were not significant, and none of the variables predicted these biomarkers (Table 1).

Finally, logistic regression using the enter method did not yield a significant model for predicting competition outcomes (χ^2^ [6] = 8.99, p = 0.17). However, in the backward stepwise model, somatic anxiety emerged as a significant predictor of competition outcomes (Table 1).

## 4 Discussion

Our results revealed a significant positive association between sCort and both somatic and cognitive anxiety. Somatic anxiety reflects physiological arousal that activates the HPA axis, increasing cortisol and potentially intensifying somatic symptoms in a feedback loop [3]. Similarly, Cognitive anxiety activates the HPA axis through threat appraisal, impairing focus and decision-making under pressure [11]. Systematic monitoring of psychological and physiological indicators in elite female karate athletes thus enables tailored interventions to optimize stress management.

No significant associations were found between anxiety subscales and sIL-6 and sTNF-α. Although stress can elevate these cytokines in clinical settings [8], cortisol's immunosuppressive feedback may downregulate them to maintain homeostasis during acute competitive stress [7,12]. This suggests that cortisol is a more reliable biomarker of competitive anxiety than inflammatory cytokines [4], whose roles appear complex and warrant further research considering timing and individual variability. Additionally, no significant associations emerged between self-confidence and salivary biomarkers, possibly reflecting the overwhelming impact of competitive anxiety, which may diminish the protective function of self-confidence under high-anxiety conditions [13]. This highlights the intricate interplay between psychological resilience and physiological stress responses.

The dissociation between salivary cortisol— despite its association with both cognitive and somatic anxiety—and actual performance outcomes underscores a critical distinction between physiological stress reactivity and the subjective experience of anxiety. Cortisol, as a biomarker of HPA-axis activation, reflects general arousal but does not capture how individuals cognitively appraise that arousal [14]. In contrast, performance is more strongly shaped by such appraisals [15]: according to the biopsychosocial model of challenge and threat, identical physiological states can yield divergent performance outcomes depending on whether the situation is perceived as a challenge (facilitative) or a threat (debilitative). Likewise, multidimensional anxiety theory posits that cognitive anxiety (e.g., worry) consistently impairs performance, whereas somatic symptoms may be neutral or even adaptive when interpreted positively [16]. Thus, self-reported anxiety likely indexes this appraisal process, rendering it a stronger predictor of performance than cortisol alone. The lack of significant predictive power from other variables may stem from biomarker fluctuations (e.g. circadian rhythm) and response delays, emphasizing the need for longitudinal and more nuanced studies.

Although direct comparisons are limited by the scarcity of studies on female karate athletes, our findings align with broader research indicating that women often report higher levels of cognitive and somatic anxiety than men in competitive settings, potentially due to socialization, perfectionism, or evaluative concerns [17]. The strong link between anxiety dimensions and cortisol in our sample may thus reflect heightened stress sensitivity among elite female athletes. Notably, in male karate athletes, some studies report weaker anxiety–cortisol associations, possibly due to differences in emotion regulation strategies or testosterone's modulatory effects on the HPA axis [4,18]. While our design does not allow sex comparisons, the robust predictive role of somatic anxiety for performance outcomes resonates with prior work in female combat sports, where bodily awareness and arousal regulation are central to success [4].

## 5 Conclusion

These findings reveal the complexity of stress regulation in elite karate athletes, where traditional predictors offer limited insight under pressure. Somatic anxiety primarily signals excessive arousal that can impair performance if unmanaged, highlighting the importance of tailored anxiety reduction and resilience training in combat sports.

## Declaration of competing interest

The authors declare that they have no known competing financial interests or personal relationships that could have appeared to influence the work reported in this paper.
